# Impact of Treatment with Direct Antivirals on Coagulation Parameters in Patients with Hepatitis C Virus-Related Liver Cirrhosis and Sustained Virological Response

**DOI:** 10.3390/medicina60091539

**Published:** 2024-09-20

**Authors:** Laura Huiban, Carol Stanciu, Cristina Maria Muzica, Irina Girleanu, Raluca Avram, Ioana Damian, Robert Nastasa, Ermina Stratina, Sebastian Zenovia, Horia Minea, Remus Stafie, Adrian Rotaru, Ana-Maria Singeap, Stefan Chiriac, Ioana-Miruna Balmus, Anca Trifan

**Affiliations:** 1 Department of Gastroenterology, Faculty of Medicine, “Grigore T. Popa” University of Medicine and Pharmacy, 700015 Iasi, Romania; huiban.laura@yahoo.com (L.H.); ralucaioanaavram@gmail.com (R.A.); robertnastasa948@gmail.com (R.N.); stratina.ermina@yahoo.com (E.S.); sebastianzenovia20@gmail.com (S.Z.); horia.minea@yahoo.com (H.M.); stafieremus@gmail.com (R.S.); adrianrotaru94@yahoo.com (A.R.); anamaria.singeap@yahoo.com (A.-M.S.); ancatrifan@yahoo.com (A.T.); 2 Institute of Gastroenterology and Hepatology, “St. Spiridon” University Hospital, 700111 Iasi, Romania; stanciucarol@yahoo.com (C.S.); ioana.galatanu@gmail.com (I.D.); 3 CENEMED Platform for Interdisciplinary Research, University of Medicine and Pharmacy “Grigore T. Popa”, 700115 Iasi, Romania; balmus.ioanamiruna@yahoo.com; 4 Department of Exact Sciences and Natural Sciences, Institute of Interdisciplinary Research, “Alexandru Ioan Cuza” University of Iasi, 700057 Iasi, Romania

**Keywords:** coagulation parameters, procoagulant factors, anticoagulant factors, chronic hepatitis C infection, direct-acting antiviral therapy, sustained virological response, liver cirrhosis

## Abstract

*Background and Objectives*: Sustained virologic responses (SVRs) lead to a decrease in portal hypertension, the regression of fibrosis, and the improvement in the hepatic synthesis of procoagulant and anticoagulant factors. We aimed to assess the influence of SVR on coagulation parameters in cirrhotic patients with HCV treated with DAAs. *Methods*: We performed a prospective study in the Institute of Gastroenterology and Hepatology Iasi, Romania, between January 2022 and February 2024. We included patients diagnosed with compensated and decompensated HCV-related liver cirrhosis, treated with direct antivirals (PrOD ± RBV or SOF/LED ± RBV) for 12/24 weeks. Blood samples for biochemical, immunological, and coagulation tests were collected at the baseline, end of treatment (EOT), and once sustained virological response had been achieved over a period of 12/24 weeks (SVR12/24). *Results*: We analyzed a group of 52 patients with HCV-related liver cirrhosis, predominantly female (68.0%), and the degree of severity of cirrhosis placed the patients mainly in Child–Pugh classes B (40%) and C (36%). All patients achieved SVRs. The MELD score decreased at EOT (13.48 ± 4.273; *p* = 0.001) and SVR (9.88 ± 2.774; *p* = 0.000), compared to the baseline (14.92 ± 4.707). The FibroScan values decreased at SVR (17.596 ± 3.7276; *p* = 0.000) compared to the baseline (26.068 ± 7.0954). For all common coagulation parameters (platelets, INR, PT, fibrinogen, aPTT), there was a trend towards improvement during treatment, including changes which were statistically significant for the majority of patients. Factor II was low at the baseline (75.40 ± 7.506) but increased at EOT (87.40 ± 9.587) and, later, at SVR (99.12 ± 11.695; *p* = 0.000). The FVIII values increased at the baseline (175.52 ± 16.414) and decreased at EOT (151.48 ± 13.703) and SVR (143.40 ± 13.937). The FvW values decreased during treatment (146.84 ± 9.428, at baseline; 141.32 ± 9.690, *p* = 0.000, at EOT; and 126.68 ± 17.960, at SVR). In regard to the anticoagulant factors (PC, PS, ATIII), a significant improvement was brought on by SVR. Advanced stages of liver disease showed the most diminished FII activity, while at the baseline and in Child–Pugh C patients we recorded the highest values of FVIII and FvW. *Conclusions*: Our study proved that the “reset” of coagulopathy might be due to the improvement in liver function due to viral eradication secondary to AAD therapy.

## 1. Introduction 

Achieving a sustained virological response (SVR) has a significant impact on the natural course of liver disease related to chronic viral C infection. In numerous clinical studies, a SVR has been shown to induce the regression of liver fibrosis, the improvement in liver synthesis, and, implicitly, the reduction in portal hypertension, which are favorable outcomes in the disease’s progression and changes highlighted primarily in patients with mild or moderate fibrosis [1,2,3]. 

Another beneficial effect of the eradication of the C virus is brought on by the “reset” of the coagulopathy, an indirect effect that reverses the hypercoagulability status, associated with the cirrhotic patient, by modifying both the procoagulant and anticoagulant factors within close quarters of the normal parameters, thus significantly lowering the hemorrhagic or thrombotic complications [4,5].

It is already known that hemostasis in a cirrhotic patient presents some features of coagulation compared to physiological hemostasis, changes which result from the status of advanced liver disease [6]. Recent studies report that the hepatitis C virus (HCV) additionally influences the coagulation status and may cause thrombotic or hemorrhagic complications to occur more frequently in patients with chronic HCV infection [7]. Inflammation at the vascular level together with altered coagulation secondary to HCV infection are elements that contribute to the increased risk of thromboembolic diseases [8]. On the other hand, thrombocytopenia and lower levels of clotting factors contribute to an increased risk of bleeding [9]. Thus, HCV infection is responsible for manifestations of both hypercoagulability and hypocoagulability [10]. 

Nielsen et al. are among the few researchers who have specifically investigated, in a prospective study, the hemostatic function of whole blood in patients with chronic HCV infection with varying degrees of liver fibrosis (absent, mild, advanced) treated and without antiviral treatment and the possible effects of viral replication on coagulation [11]. 

The effect of HCV viral replication on coagulation parameters was evaluated in the same prospective study involving patients treated with direct antivirals and SVR [11]. Minor changes in standard post-treatment coagulation parameters were found. Post antiviral treatment, the platelet count and fibrinogen parameters showed a partial “restoration” but at lower values than the control group. The same effect was noticed for the concentration of coagulation factors II–VII–X. Thus, the question arises as to whether the change in coagulation parameters in chronic HCV infection is only a result of viral replication or whether these changes are secondary to the liver fibrosis that persists in patients even after SVR is obtained.

The impact of DAAs on coagulation parameters in patients with HCV-related liver cirrhosis is a controversial and intensely studied topic, the realm of which is still not fully explored. Among the first studies to evaluate coagulation parameters during DAAs treatment in patients with HCV infection was the one conducted by Tripodi et al. in 2017 [4], which investigated coagulation status in patients infected with HCV before, during, and after treatment, using traditional global and individual coagulation tests, as well as state-of-the-art methods, including thrombin generation with and without thrombomodulin, and thromboelastometry as a global method of coagulation status analysis. The authors of the study demonstrated, in a prospective study, the beneficial effect of DAAs on pro- and anticoagulant factors, by improving pro- and anticoagulant status; DAAs do not substantially alter their balance, but make them more stable and less likely to be disrupted, as assumed before treatment [4].

Data from the literature show that obtaining an SVR leads to improved hepatic synthesis of both procoagulant and anticoagulant factors and, implicitly, a slow recovery of HCV-induced coagulopathy [4,5,12]. At the same time, recent studies have shown that obtaining an SVR in patients with HCV-related liver cirrhosis is associated with improved MELD and Child–Pugh scores, which reflect improved hepatic synthesis function and, implicitly, the synthesis of coagulation factors [13,14,15,16,17,18,19,20,21]. 

The main objective of this study was to evaluate how coagulation parameters are influenced by the SVR obtained in patients with HCV cirrhosis, treated with AAD therapy, and the correlation between the coagulation factors and liver cirrhosis severity. 

## 2. Materials and Methods 

### 2.1. Patients

We included randomly selected patients diagnosed with compensated and decompensated HCV-related liver cirrhosis who were eligible, in accordance with the Ministry of Health’s criteria, to be treated with direct antivirals (ombitasvir/paritaprevir/ritonavir + dasabuvir ± ribavirin or sofosbuvir/ledipasvir ± ribavirin) for 12/24 weeks. The diagnosis of cirrhosis was based on clinical, biological, and imaging data according to international guidelines. 

The diagnosis of viral C infection was suspected via anti-HCV Ab positivity and confirmed by HCV-RNA, the detection limit being 15 IU/mL. A sustained virologic response was defined as an undetectable HCV-RNA level 12/24 weeks after the completion of direct antiviral treatment. This study excluded patients with an age of less than 18 years, pregnant or breastfeeding women, patients with human immunodeficiency virus defined by positive anti-HIV antibodies, liver cancer without an indication for transplantation, treated by ablation or resection less than 6 months after the procedure, or with CT/MRI signs of post-procedure tumor recurrence, and patients with other extra-digestive neoplasms, thrombophilia, anticoagulant treatment, chronic ethanol consumption, or drug contraindications for DAA therapy.

### 2.2. Study Protocol

**Figure 1:**
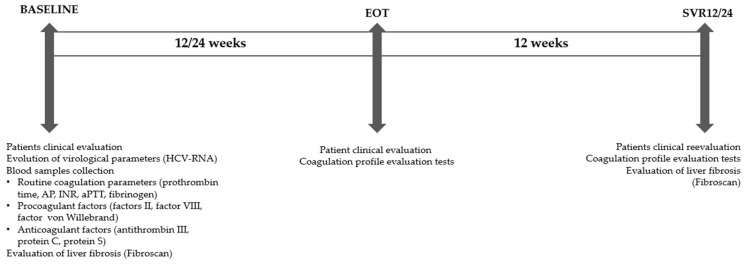
Flowchart of the study protocol.

We performed a prospective study at the Institute of Gastroenterology and Hepatology Iasi, Romania, between January 2022 and February 2024. We collected blood samples at 3 moments in time: at the initiation of antiviral treatment (baseline), at the end of treatment (EOT), and 12 weeks after the completion of antiviral treatment (SVR12/24) (Figure 1). 

#### 2.2.1. Laboratory Assessment

Blood samples for the coagulation tests were collected from the antecubital vein. Two vacutainers of whole blood containing 1/10 volume of sodium citrate as the anticoagulant were taken, each vacutainer having a capacity of 2.7 mL. The blood samples were centrifuged for 15 min at 2500× *g*/min, and the plasma was collected and stored in 0.5 mL devices and subsequently frozen at −80 °C until examination. The processing of the biological samples was performed after thawing the plasma at 37 °C for 5 min. Hematological measurements were performed from blood obtained on the same day of blood collection at the time of the medical visit. 

#### 2.2.2. Coagulation Profile Evaluation Tests

Prothrombin time, AP, INR, aPTT, and fibrinogen were determined by the coagulometric method using an automatic analyzer according to the instructions (Sysmex CA—CA-600, SYSMEX CORPORATION, 1-5-1 Wakinohama-Kaigandori, Chuo-ku, Kobe 651-0073, Japan). The activity of factors II and VIII was determined by the coagulometric method, with the help of an automatic analyzer (ACL TOP 750, Instrumentation Laboratory, Bedford, MA, USA). The von Willebrand factor activity measurement was performed by the immunoturbidimetric method, using the same analyzer (ACL TOP 750). The anticoagulant and chromogenic activity of AT III and protein C was determined using the automatic analyzer ACL TOP 750, and protein S was determined using the coagulometric method. The activity of all factors was determined using the following kits: Bedford, MA, 01730-2443 (USA); and Monza, 338-20128, Milano (Italy).

### 2.3. Evaluation of Liver Fibrosis

To assess liver fibrosis as part of our inquiry, we utilized the FibroScan^®^ 520 compact model (Echosens, Paris, France) that comes with an M (normal) probe with a 3.5 MHz transducer frequency or an XL (obese) probe with a 2.5 MHz transducer frequency. The patients were instructed to maintain a supine position, with the right arm fully extended, after at least 4 h of fasting. At first, the M probe was used for the investigation. However, if the distance between the liver capsule and the skin exceeded 25 mm, the XL probe was used. A reliable measurement was defined by 10 acquisitions within the cutoff of 30% for the interquartile interval. We defined mild fibrosis (F1) at a liver stiffness measurement between 5.6 and 7.1 kPa, significant fibrosis between 7.1 and 9.4 kPa (F2), advanced fibrosis between 9.5 and 12.4 kPa (F3), and cirrhosis over 12.5 kPa (F4) [22]. 

### 2.4. Statistical Analysis

For the statistical analysis, Microsoft Excel (Microsoft® Excel® 2019 MSO (Version 2408 Build 16.0.17928.20114)) and SPPS (Inc., Chicago, IL, USA, version 28) were the programs used. There were 2 kinds of variables that occurred: categorical variables, which were identified in the form of absolute values and percentages, while continuous variables with normal distributions were in the form of the mean SD. The statistical equations and tests used were as follow: Chi-square for categorical data comparison, *t*-Student test for the arithmetic means of a parameter analyzed in two-sample comparison, the Mann–Whitney U test for non-parametric tests, and the Kolmogorov–Smirnov test in order to assess data distributions. The statistical significance of the results was illustrated by a *p*-value of 0.05.

### 2.5. Ethical Considerations

The ethical principles of the declaration of Helsinki were respected, with the study protocol being signed and explained in detail to all the patients. Informed consent for study inclusion was obtained from all the patients, thus agreeing to additional monitoring and blood sampling during antiviral treatment. 

## 3. Results

### 3.1. General Characteristics of the Study Population

We analyzed a group of 52 patients with HCV-related liver cirrhosis, who were predominantly female (68%), with an average age of 63.88 ± 9.387 years; the degree of severity of cirrhosis placed the patients mainly in Child–Pugh classes B (40%) and C (36%). All patients achieved an SVR. None of the patients developed complications during the study period.

On average, the MELD score had a value of 14.92 ± 4.707, with more than half of the patients (60%) having a MELD ≥ 15 compared to the others (40%). Most patients included in this study (76%) received treatment with LED/SOF, while 15 patients (28%) also received the associated RBV, and most patients—37 (72%)—were naïve to antiviral treatment (Table 1).

### 3.2. Evolution of Virological Parameters, Liver Function, and the Degree of Liver Fibrosis in the Studied Group

Undetectable HCV-RNA at EOT and SVR was achieved by all patients. The MELD score registered a statistically significant decrease at the EOT (13.48 ± 4.273; *p* = 0.001), and, at the time of SVR, an even more pronounced decrease was observed (9.88 ± 2.774; *p* = 0.000), compared to values measured at the initiation of antiviral treatment (14.92 ± 4.707). Fibroscan values were recorded only at the initiation of antiviral treatment and at the time of SVR, where a statistically significant decrease was observed (17.596 ± 3.7276; *p* = 0.000) compared to the baseline value (26.068 ± 7.0954). 

### 3.3. Evaluation of Routine Coagulation Parameters in Patients with HCV-Related Liver Cirrhosis Treated with DAAs and SVR

For all common coagulation parameters, there was a trend towards improvement during antiviral treatment, with changes which were statistically significant for the majority of patients included in this study. 

**Figure 2:**
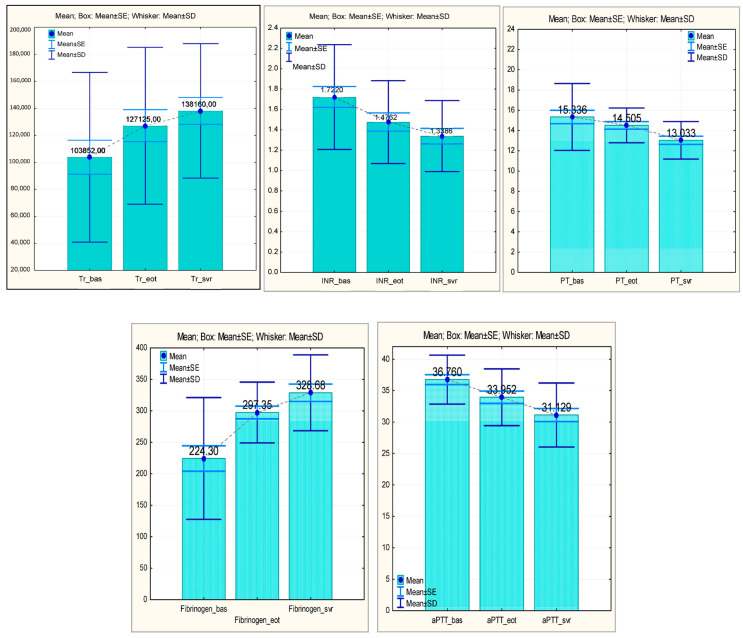
Usual coagulation parameters (platelet, INR, PT, fibrinogen, and aPTT)—evolution during antiviral treatment (baseline, EOT, and SVR). Tr, thrombocytes; INR, international normalized ratio; PT, prothrombin time; and aPTT, activated partial thromboplastin time.

In the studied group, it was observed that the initial average value of platelets (103,852.00 ± 62,837.138) registered a statistically significant increase both at EOT (127,125.00 ± 58,090.868, *p* = 0.005) and at SVR (138,160.00 ± 49,819.407). The initial mean INR value (1.7220 ± 0.51522) decreased statistically significantly at EOT (1.4762 ± 0.40713; *p* = 0.001), and, at the time of SVR, the decreasing trend was maintained (1.3386 ± 0.34864; *p* = 0.001). The initial mean value of PT (15.336 ± 3.3041) decreased statistically significantly both at EOT (14.505 ± 1.7142; *p* = 0.026) and at SVR (13.033 ± 1.8529; *p* = 0.001). The fibrinogen values also registered a statistically significant increase, constant over time—224.30 ± 96.803 at the baseline, 297.35 ± 48.295 at EOT, and reaching the average value of 328.68 ± 60.178 at SVR. A similar trend in variation was also observed in the case of aPTT, where a statistically significant decrease (*p* = 0.004) was found in the values at EOT (33.9519 ± 4.51637) and SVR (31.1290 ± 5.09690; *p* = 0.000) compared to the baseline (36.760) (Figure 2). 

### 3.4. Evaluation of Procoagulant Factors in Patients with HCV-Related Liver Cirrhosis Treated with DAAs and SVR

When the AAD treatment was first started, blood tests showed that the activity of factor II was decreased and that of factor VIII and vW was increased. Characteristically of patients with liver cirrhosis, an increase in factor VIII was reported, which is also prevalent in the case of portal vein thrombosis. 

In our study, at admission, the mean value of FII was low (75.40 ± 7.506), increasing with statistical significance (*p* = 0.000). At EOT, it reached a mean of 87.40 ± 9.587, followed by a maintained improvement at SVR, still remaining statistically significant, with a final mean FII of 99.12 ± 11.695 (*p* = 0.000).

At the same time, the FVIII values increased upon the initiation of the antiviral treatment (175.52 ± 16.414) and decreased statistically significantly at the time of EOT (151.48 ± 13.703) and SVR (143.40 ± 13.937). 

**Figure 3:**
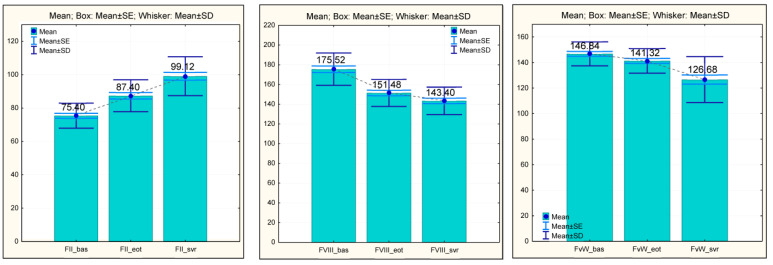
Procoagulant factors (FII, F VIII, and FvW)—evolution during antiviral treatment (baseline, EOT, and SVR). FII, factor II; F VIII, factor VIII; and FvW, factor von Willebrand.

The FvW followed a similar trend, where a constant decrease in values was observed during antiviral treatment. At the baseline, a mean FvW value of 146.84 ± 9.428 was measured, which, at EOT, decreased statistically significantly (141.32 ± 9.690; *p* = 0.000). At SVR, the final mean value of FvW (126.68 ± 17.960) further decreased statistically significantly (*p* = 0.000) compared to the initial assessment (Figure 3). 

### 3.5. Evaluation of Anticoagulant Factors in Patients with HCV-Related Liver Cirrhosis Treated with DAAs and SVR

In our study, all anticoagulant factors, such as protein C, protein S, and antithrombin III, had low values upon the initiation of antiviral therapy. This certified the hemostatic imbalance, with a predisposition towards the occurrence of thrombotic accidents in the case of cirrhotic patients. A significant improvement in the anticoagulant factors’ values in patients with SVR was noted, similarly to the evolution of the procoagulant factors.

The PC values increased statistically significantly (*p* = 0.000) from the initial evaluation (68.92 ± 8.411) to the EOT (86.24 ± 12.879), a phenomenon which was also observed at the time of SVR evaluation (95.84 ± 11.898).

The PS values evolved in the same way, having a low mean value upon the initiation of treatment (68.44 ± 3.163), increasing statistically significantly (*p* = 0.000) at EOT (88.48 ± 15.951), accentuating the increase at the time of SVR, with the average value of PS reaching 95.64 ± 15.327 (*p* = 0.000).

**Figure 4:**
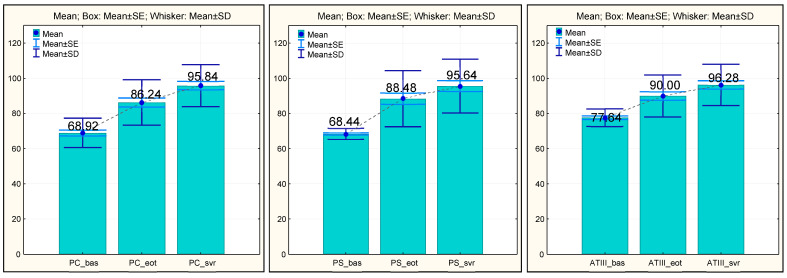
Anticoagulant factors (PC, PS, and AT III)—evolution during antiviral treatment (baseline, EOT, and SVR). PC, protein C; PS, protein S; and AT III, antithrombin III.

The ATIII values increased steadily over time, in a manner which was also statistically significant (*p* = 0.000). At the initial assessment, the low mean value was 77.64 ± 4.999 and registered an increase at the EOT (90.00 ± 11.969), reaching a significant value at the time of SVR (96.28 ± 11.778) (*p* = 0.000) (Figure 4).

### 3.6. Evaluation of the Relationship between the Severity of Liver Cirrhosis and Changes in the Coagulation Factors

**Figure 5:**
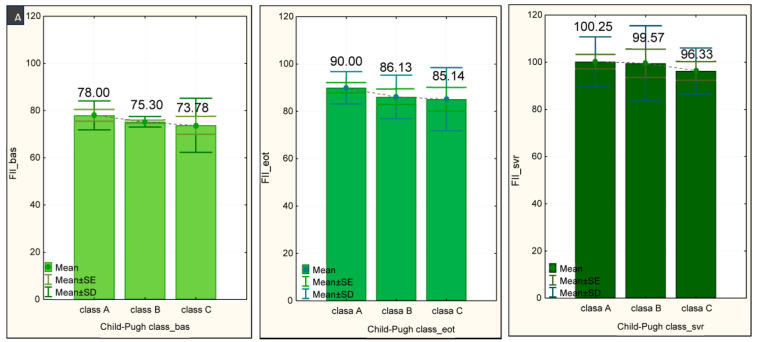

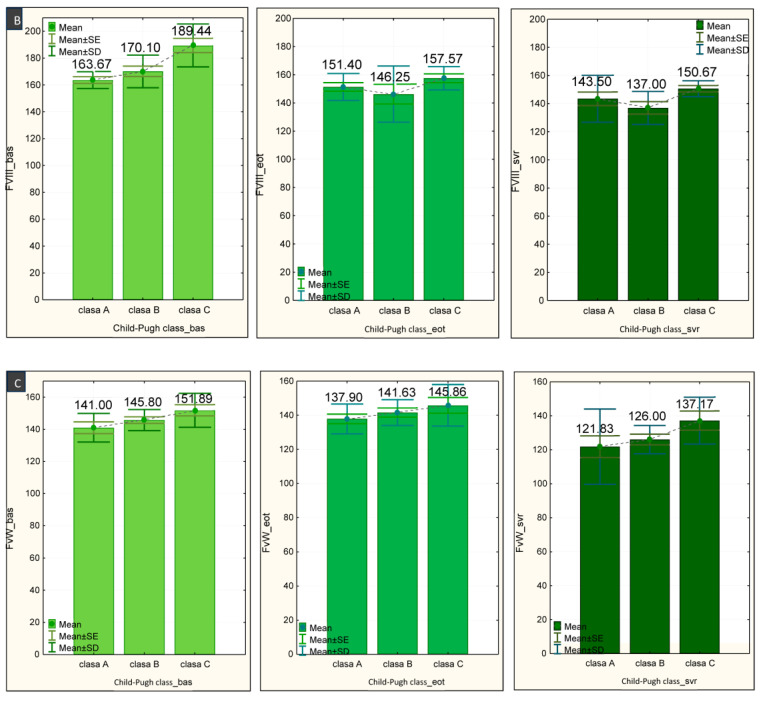
Procoagulant factors—initial value and evolution of values over time, compared by Child–Pugh classes. (**A**) Factor II—the evolution according to the Child-Pugh class; (**B**) Factor VIII—the evolution according to the Child-Pugh class; (**C**) Factor vW—the evolution according to the Child-Pugh class.

During the time allotted for this study, the evolution of coagulation factors in correlation to liver damage severity from the baseline to the EOT and, finally, the SVR was investigated. The most advanced stages of liver disease showed the most diminished FII activity, with no statistically significant differences between the three Child–Pugh classes during the follow-up period (Figure 5A). At the baseline, in Child–Pugh class C patients, we recorded the highest FVIII (Figure 5B) and FvW (Figure 5C) values.

Throughout this study, the PC differences between Child classes showed statistical significance (*p* = 0.001). Therefore, the PC values were increased statistically significantly at the time of SVR compared to the initial assessment for all three severity classes; patients in Child–Pugh class C had the least increase in PC compared to patients in Child–Pugh classes A and B (Table 2A). 

Similarly, the protein S values were lowest at the initial evaluation, with statistical significance, among the three classes, in patients with Child–Pugh C (*p* = 0.002). Afterwards, the PS values increased at the EOT assessment compared to the initial values, showing no statistically significant difference between the three Child classes (*p* = 0.665). At the time of SVR evaluation, the PS values continued to increase, again without statistical significance (*p* = 0.781) in the differences between the three Child classes, with Child–Pugh C class patients having slightly lower mean PS values compared to the other patients (Table 2B). 

**Figure 6:**
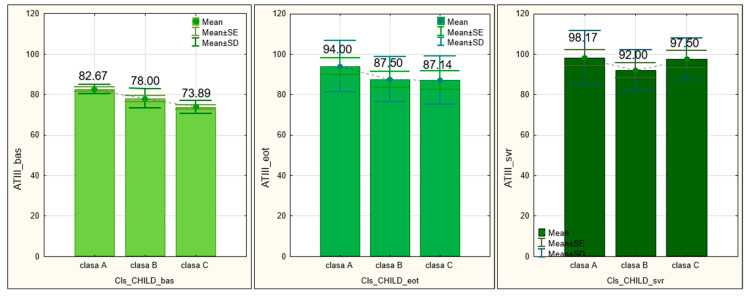
Antithrombin III’s initial value and evolution of values over time, compared by Child–Pugh classes.

Finally, the lowest ATIII values were observed at the baseline, where a significant statistical difference between patients in various Child classes was found (*p* = 0.001). Thus, the ATIII values were slightly higher in class A patients, whereas patients from class B and class C had statistically significantly lower ATIII values. At EOT, the ATIII values increased slightly and became uniform between the three Child classes, the differences recorded lacking statistical significance (*p* = 0.438). The highest values were observed in patients belonging to Child A, while patients in Child B and C had close values. The ATIII values continued to increase also at the time of SVR assessment, where no statistically significant differences were recorded between the three Child classes (*p* = 0.449). The highest values of ATIII were observed in the patients in the Child A class, while the lowest values of ATIII were observed in the patients in the Child B class, with the patients in the Child C class recording intermediate values, close to those of the patients in the Child A class (Figure 6).

## 4. Discussion

The natural course of HCV-related liver disease is significantly impacted by achieving a sustained virological response, due to the improvement in liver function which results from direct antiviral therapy, effectively bringing the levels of coagulation factors within the normal range, causing a “reset” [5]. 

The present study showed that viral eradication due to AAD therapy improved liver function by bringing some of the coagulation factors within the normal range, thus resolving the hypercoagulable state.

Among the first studies to evaluate coagulation parameters during DAA treatment in HCV-infected patients was that by Tripodi et al. in 2017, who demonstrated, in a prospective study, the beneficial effect of AAD on pro- and anticoagulant factors [4]. The authors investigated the coagulation status of HCV-infected patients before, during, and after treatment using traditional global and individual coagulation tests as well as state-of-the-art methods, including thrombin generation with and without thrombomodulin, whose role had been previously shown to depict coagulation much more realistically than conventional tests. In addition, thromboelastometry was also used as a global method for analyzing the coagulation status, which graphically translates the entire process of clot formation and continues, even after its formation, with the evaluation of clot lysis and retraction—the fibrinolytic phase. Thus, it was possible to analyze the viscoelastic characteristics of blood, properties which have already been shown to be abnormal in cirrhotic patients. The prothrombin time, thrombin generation with or without thrombomodulin, thromboelastometry, and procoagulant (II, VIII, XIII, and von Willebrand) and anticoagulant (antithrombin III and protein C) factors were analyzed.

In the present study, the investigated group consisted of patients with viral liver cirrhosis C, the majority being female (68%); the degree of severity of cirrhosis placed the patients mainly in Child–Pugh classes B and C, a fact which attests to the heterogeneity of the study group, which consisted of both compensated and decompensated cirrhotic patients, with an advanced degree of liver fibrosis.

Most of the patients included in this study, i.e., more than three quarters of them (76%), received treatment with LED/SOF, as this was the only one recommended and available at the time in our country for the treatment of patients with decompensated HCV or at the limit of hepatic decompensation.

In our study, the MELD score values decreased significantly at EOT and SVR. These results are consistent with previous reports that also demonstrated a significant decrease in the liver parameters in patients attaining SVR [23]. Negro et al. reported that viral clearance is often associated with fewer liver decompensation events and, in more than half of the patients, lower MELD scores [24]. However, Krassenburg LAP et al. showed that patients with Child–Pugh B/C cirrhosis had a mild decrease in their MELD scores after AAD therapy, suggesting that viral eradication was attainable, yet liver damage persisted [25]. In these patients (almost 20%), the decrease in the MELD score was by at least two points 12 weeks after the end of therapy. A more recent study performed on patients with HCV from the USA waiting for liver transplantation reported that, ever since the availability of DAAs to treat these patients, the slope of the MELD score changed, and the survival of the patients in the cohort receiving DAAs improved [26]. Also, they found that the ΔMELD between the cohorts not receiving DAAs and those receiving treatment was significantly decreased [26]. One of the largest studies, including 409 patients with decompensated cirrhosis treated with AAD, showed that more than 80% of treated patients achieved SVR12, and a mean change of −0.85 in the MELD was obtained, suggesting viral eradication and improvement in liver functions. By comparison, the mean change in the MELD in the untreated patient group suggested a worsening of liver functions during the period of observation [27]. However, the study also mentioned that the sustained viral response status at 12 weeks could not predict MELD score changes.

All patients included in this study had undetectable HCV RNA at EOT and SVR. The dramatic decrease in viremia after antiviral treatment also observed in this study was consistent with data from the literature [28], demonstrating that we included a cohort of patients that could be adapted to any population of cirrhotic patients with HCV infection who are candidates for direct antiviral treatment.

In this study, we observed the dynamic changes over time of a wide range of coagulation parameters from the patients’ first presentation to the EOT and, finally, upon their achievement of an SVR. Traditional coagulation tests determining measures such as platelet, INR, PT, and aPTT values, and individual coagulation factors such as procoagulant factors FII, FVIII, and FvW (known to illustrate important changes in cirrhotic patients) and anticoagulant factors (antithrombin III, protein C, and protein S) were monitored during this time period. 

Studies in recent years have supported the idea that the coagulopathy reflected, in particular, by the increase in aPTT, INR, and prothrombin is associated with decreased liver synthesis function. The prothrombin time is a prognostic factor for survival, being an important marker of liver failure [29].

In the studied group, cirrhotic patients, at the time of initiating AAD therapy, presented with a hypocoagulant status, diagnosed by increased INR, prothrombin time, and aPTT, associated with fibrinogen values within the normal range. These normal values of fibrinogen showed the existence of the basic substrate for thrombin’s action, and so, all coagulation parameters showed a statistically significant trend towards improvement during antiviral therapy for most of the patients included in this study.

There was a statistically significant increase in the baseline mean platelet count at the end of treatment, a trend which was maintained in both directions and with statistical significance (*p* = 0.000), and at the time of SVR, when the highest platelet counts were achieved. 

The results of our study are consistent with those reported by Koh et al. [30]. By prospectively following 100 patients for 23 months post SVR, they reported significant improvements in the platelet counts of patients who achieved SVR. Moreover, no altered prothrombin time was reported post SVR, while the platelet counts increased, falling within the normal range in approximately 90% of patients who had achieved SVR, disregarding fibrosis severity, but in an inversely proportional correlation with the degree of liver stiffness [30].

Regarding this aspect, in contrast with our results, Tripodi et al. [4] found that the platelet counts showed no significant improvement at EOT or SVR, despite all other coagulation parameters. Additionally, they reported that the prothrombin time non-significantly decreased at EOT and SVR12, compared to the baseline, which is also an important contrasting result to our data. On the other hand, another recent study performed by Nielsen et al. [11] reported only minor changes in standard coagulation parameters post treatment. In this way, at the time of SVR, platelet counts and fibrinogen were partially “restored” (still, with lower mean values compared to the control group). Similar trends were observed for factors II, VII, and X, in which improvement post SVR was noted. Meanwhile, in our study, the FII significantly increased post SVR, compared to the baseline, reaching a normal physiological range. 

Nevertheless, we found that the mean values of the most common coagulometric parameters were correlated with the Child–Pugh score at the baseline, EOT, and SVR12/24, suggesting an inverse proportionality between their values and the severity of liver damage. Nielsen et al. [11] were among the few researchers who specifically prospectively investigated the hemostatic function of whole blood in patients diagnosed with chronic HCV infection and liver fibrosis (absent, mild, and advanced), both with and without antiviral treatment, and the possible effects of viral replication on coagulation [11]. Compared to the control group of healthy patients, patients with chronic HCV infection with both mild and advanced fibrosis had thrombocytopenia and low antithrombin III concentrations, predisposing them to thrombosis. At the same time, an increase in platelet aggregation was observed in patients with advanced fibrosis compared to those without or with mild liver fibrosis, a phenomenon which explains the occurrence of thrombotic events in patients with chronic HCV infection and increased fibrosis. At the same time, the fibrinogen and aPTT values were decreased in the case of HCV infection, but their values did not differ based on the degree of liver fibrosis [11].

At the same time, recent studies that demonstrate an improvement in the number of platelets after AAD treatment raise an alarm in the case of this patient population, with this phenomenon being considered a predictive factor for triggering a thrombotic event [31,32,33,34]. Campello E et al. [35], in a prospective study which included 58 patients with HCV cirrhosis treated with DAAs, mention that an increase in the number of platelets post SVR is, at the same time, a risk factor for the occurrence of thrombotic complications in patients with more severe liver disease and portal hypertension. In the studied group, the authors reported three patients with Child B who developed non-malignant PVT one year after the end of antiviral treatment [35]. During our study, none of the patients developed thrombotic or hemorrhagic complications, but the follow-up period was short.

In the studied group, it was noted that the activity of all procoagulant factors was modified upon the initiation of antiviral treatment. The activity for factor II was decreased, while increased values of the activity of factor VIII and factor von Willebrand were recorded, painting a picture which defines the hypercoagulable status of cirrhotic patients at risk of thrombotic accidents. But, over the course of this study, a statistically significant improvement in the SVR was observed in these factors, with increasing FII and decreasing factor VIII values, alongside factor von Willebrand, which followed a similar trend.

Similar to the evolution of the procoagulant factors, in the case of anticoagulant factors (PC, PS, and ATIII), a significant improvement in values was noted in patients with a sustained virological response. The PC, PS, and AT III values systematically increased over time, from the initial assessment (characteristic of a hypocoagulant status) to the other two determinations, reaching a significant value at the time of SVR. The exact reasons for the reduction in procoagulant imbalance are not definitively known, but the reduction in factor VIII concomitant with the increase in protein C following AAD treatment may be a likely explanation. Moreover, the simultaneous increase in AT III after treatment may have contributed to the reduction in this procoagulant imbalance.

The results of clinical trials have shown that high levels of factor vW are a distinct feature of patients with liver cirrhosis. In this study, we demonstrated that factor vW decreased in patients treated with antivirals, a fact which can be considered secondary to the beneficial effect of this therapy.

The results of our study are confirmed by those obtained by Tripodi et al. [4], who demonstrated that FII, PC, and AT III increased significantly at EOT and persisted at the time of SVR. Factor VIII’s concentration registered a statistically significant (*p* < 0.005) progressive decrease from EOT to SVR. Factor vW’s level decreased at the end of treatment, reaching statistical significance at the time of SVR.

Russo et al. obtained the same results [5], showing that a decrease in FVIII and an increase towards normal values for protein C were the most significant changes in the coagulation parameters. Moreover, an increase in FII, AT III, and PS highlighting the improvement in pro- and anticoagulant factors’ liver synthesis in patients having achieved SVR was demonstrated. On the other hand, Nielsen et al. [11] observed that patients with chronic HCV infection treated with AAD and SVR had a partial normalization of coagulation factors II, VII, and X.

The mechanisms through which DAAs exert an effective action in ameliorating pro- and anticoagulant factors’ functions in patients with HCV attaining SVR are partly understood. On the one hand, it is currently widely accepted that the synthesis of coagulation factors is impaired in cirrhotic patients. However, similar impairments could be observed due to accelerated viral clearance in patients achieving SVR. According to this hypothesis, viral-promoted pro-inflammatory cytokines’ activation could lead to the increase in coagulation factors’ clearance from the bloodstream [36]. In this way, HCV’s implications in the coagulation mechanisms of patients could be direct, decreasing liver synthesis capacity, and/or indirect, accelerating the clearance of coagulation factors. On the other hand, a prospective study on 58 HCV patients receiving DAAs recently reported the correction of hypercoagulable states post SVR [5]. However, in the absence or with a low number of moderate and severe cases, the authors concluded that, in mild cirrhotic patients (Child A), DAAs treatment could lead to the amelioration of the liver synthesis of coagulation factors and inhibitors and the subsequent correction of the anticoagulant protein C system [5]. Also, in a recent study, Meissner et al. [37] showed that there is a normalization of cytokines in patients with HCV infection treated with DAAs, but the latter’s effect on coagulation factors was not studied.

## 5. Conclusions

The correlation between the recently emerged DAA treatments for HCV eradication and the coagulation states in patients with chronic viral liver disease could be a promising hypothesis. Despite the increasing body of evidence, DAAs are still relatively new antiviral therapies, lacking long-term follow-up observation. Thus, the improvement in coagulation states in patients with HCV post SVR could be the effect of multiple mechanisms, including yet-undescribed antiviral treatment mechanisms of action or the subsequent improvement and stabilization of liver functions, as shown by both our data and most previous clinical trials in which viral eradication was obtained.

## Figures and Tables

**Table 1:** Baseline characteristics of the study patients.

Parameters	Patients
	N = 52
Sex	
male, n (%)	17 (32%)
female, n (%)	35 (68%)
Age (years)	63.88 ± 9.387
Child–Pugh class	
class A, n (%)	12 (24%)
class B, n (%)	21 (40%)
class C, n (%)	19 (36%)
MELD score	
<15	21 (40%)
≥15	31 (60%)
Treatment DAA	
PrOD, n (%)	12 (24%)
LED/SOF, n (%)	40 (76%)
Experienced/naïve to IFN, n (%)	15 (28%)/37 (72%)
Ribavirin, n (%)	15 (28%)
Antecedents of decompensation, n (%)	29 (56%)
History of esophageal varices, n (%)	35 (68%)
Fibroscan, kPa	26
SVR, n (%)	52 (100%)

n, number of subjects; SVR, sustained virologic response; DAA, direct-acting antivirals; PrOD, ombitasvir/paritaprevir/ritonavir, dasabuvir; LED, ledipasvir; SOF, sofosbuvir; IFN, interferon; and kPa, kilopascal.

**Table 2:** (**A**) Protein C’s initial value and evolution of values over time, compared by Child–Pugh classes. (**B**) Protein S’s initial value and evolution of values over time, compared by Child–Pugh classes.

(**A**)			
**Protein C**			
**CHILD Class**	**N**	**Mean ± SD**	***p*-Value**
Baseline			0.001
class A	14	75.50 ± 3.146	
class B	20	71.30 ± 3.129	0.075
class C	18	61.89 ± 9.993	0.010
EOT			0.002
class A	22	87.30 ± 10.253	
class B	17	95.50 ± 10.433	0.102
class C	13	74.14 ± 9.582	0.015
SVR			0.164
class A	24	96.75 ± 11.395	
class B	16	100.71 ± 12.392	0.474
class C	12	88.33 ± 10.328	0.156
(**B**)			
**Protein S**			
**CHILD Class**	**N**	**Mean ± SD**	***p*-Value**
Baseline			0.005
class A	13	71.83 ± 2.787	
class B	21	67.60 ± 2.011	0.005
class C	18	67.11 ± 3.018	0.002
EOT			0.665
class A	20	90.10 ± 3.018	
class B	19	90.63 ± 19.835	0.947
class C	13	83.71 ± 10.484	0.437
SVR			0.781
class A	25	96.75 ± 15.184	
class B	15	97.14 ± 18.898	0.959
class C	12	91.67 ± 12.910	0.527

## Data Availability

The data presented in this study are available upon request from the corresponding author. The data are not publicly available because they are the property of the Institute of Gastroenterology and Hepatology, Iasi, Romania.
